# Multi-Walled CNTs/BiVO_4_ Heterostructures for Solar-Driven Evaporation System and Efficient Photocatalytic Activity against Oxytetracycline

**DOI:** 10.3390/nano13202764

**Published:** 2023-10-15

**Authors:** Muneerah Alomar, Naila Arshad, Muhammad Sultan Irshad, Shaimaa A. M. Abdelmohsen, Iftikhar Ahmed, Nawal Alhoshani, Areej S. Alqarni

**Affiliations:** 1 Department of Physics, College of Sciences, Princess Nourah bint Abdulrahman University, P.O. Box 84428, Riyadh 11671, Saudi Arabia; shamohamed@pnu.edu.sa (S.A.M.A.); nialhoshani@pnu.edu.sa (N.A.);; 2 Ministry of Education Key Laboratory for the Green Preparation and Application of Functional Materials, Hubei Key Laboratory of Polymer Materials (Hubei University), Collaborative Innovation Center for Advanced Organic Chemical Materials Co-Constructed by the Province and Ministry, School of Materials Science and Engineering, Hubei University, Wuhan 430062, China; nailasehar371@gmail.com; 3 Energy Research Centre, COMSATS University, Lahore Campus, Islamabad 54000, Pakistan; dr.iftikhar.ahmed@live.fr

**Keywords:** solar evaporation, BiVO_4_, MWCNTs, photocatalytic, freshwater, oxytetracycline

## Abstract

Developing a sustainable environment requires addressing primitive water scarcity and water contamination. Antibiotics such as oxytetracycline (OTC) may accumulate in the environment and in the human body, increasing the risks to the ecosystem. The treatment of polluted water and the production of potable water can be achieved in a variety of ways, including photodegradation, solar distillation, and filtration. Freshwater supplies can be increased by implementing energy-efficient technologies for the production of clean water. Solar water evaporation combined with photocatalytic degradation and sterilization offers a promising avenue for integration into the clean water and energy production fields. The present study reports the synthesis of a 3D solar steam generator comprised of BiVO_4_ and carbon nanotubes (CNT) nanocomposite decorated over a cigarette filter as the light-to-heat conversion layer for solar steam generation. The BiVO_4_@CNT-based 3D solar evaporator over the hydrophilic cellulosic fibers of the cigarette filter endowed excellent evaporation rates (2.36 kg m^−2^ h^−1^) under 1 kW m^−2^ solar irradiation, owing to its superior hydrophilicity and broadband solar absorption (96%) equipped with localized heating at microscale thermal confinement optimized by the minimum thermal conductivity of the overall system. Furthermore, the BiVO_4_@CNT composite exhibited a heightened photo activity up to 83% of the photodegradation of oxytetracycline (OTC) antibiotic due to the inhibition of charge recombination from the industrial effluents. This approach transforms the water-energy nexus into a synergistic bond that offers opportunities to meet expected demand, rather than being competitive.

## 1. Introduction

Despite the development of the global economy and society, freshwater scarcity will continue to pose a formidable challenge. Water pollution can exacerbate this situation due to the presence of hazardous organic pollutants in wastewater, which poses further threats to the environment and human health [1,2,3]. However, antibiotics are utilized for disease prevention in both human beings and animals and have gained increased scrutiny in recent times due to their severe environmental impact. Oxytetracycline (OTC), a member of tetracycline antibiotics, is characterized by a complex structure composed of four interconnected rings and possesses multiple functional groups that are capable of undergoing ionization. OTC antibiotics are notoriously difficult to filter out using conventional water treatment methods, posing a threat to global water supplies, and large amounts are deposited in streams and soils [4,5]. The availability of safe, reliable, and affordable water has become one of the most pressing problems these days which is anticipated to become even more prevalent in the future. According to the World Water Development Report 2020 published by the United Nations, a staggering 3.9 billion individuals are presently enduring acute physical water scarcity for a minimum duration of one month annually due to expeditious industrialization and urbanization [6,7]. Substantial efforts have been invested in exploring green technologies for clean water production. Solar-driven water evaporation, which harnesses sunlight as a sustainable energy source, presents a viable strategy for addressing the issue of water scarcity while minimizing adverse environmental effects [8,9,10]. Nonetheless, the practical implementation of solar steam generation is hindered by the limited photothermal conversion efficiency, which is primarily attributed to the inadequate solar absorption of water and the heat losses associated with traditional bulk water heating methods [11,12]. A multifunctional photothermal material can enhance the efficiency and effectiveness of solar evaporation by incorporating a multifunctional photothermal material.

Solar absorbance capabilities of multifunctional photothermal materials allow them to efficiently absorb a wide spectrum of sunlight [9,13,14]. This process absorbs solar energy and converts it into heat, which raises the temperature of the material and the water in its vicinity. The doping of metals or nonmetals or the use of other semiconductors for heterojunction development have been extensively reported to date [15]. A wide variety of studies have been conducted to enhance photocatalytic performance as semiconductor photocatalysts that are capable of responding to visible light [16,17,18]. Bismuth vanadate (BiVO_4_) has gained significant popularity as a semiconductor photocatalyst mainly because of its small band gap, low toxicity, and excellent stability, rendering it highly responsive to visible light [19,20,21]. In recent times, there has been a significant focus on investigating the potential of monoclinic BiVO_4_ in the field of visible-light photocatalysis for the degradation of organic pollutants. This interest stems from the monoclinic phase of BiVO_4_ having a lower theoretical band gap (2.4 eV) compared to the tetragonal phase (2.9 eV) of BiVO_4_ [21,22,23]. Nevertheless, there has been considerable interest in multi-walled carbon nanotubes (MWCNTs), owing to their substantial specific surface area, abundant adsorption sites, and favorable electrical conductivity. In recent years, several studies have examined the complex decomposition process of oxytetracycline antibiotics and shed light on the antibiotic’s stability, degradation pathways, and environmental impact [24]. The widespread use and subsequent release of these antibiotics into the environment makes them widely used both in human medicine as well as veterinary medicine [25]. Research has shown that oxytetracycline antibiotic degradation is affected by pH, temperature, light exposure, and coexisting compounds [26]. Hydrolysis, photolysis, and oxidation products reveal the complex transformation mechanisms of these antibiotics [27]. BiVO_4_@CNTs (Bismuth Vanadate decorated with Carbon Nanotubes) are multifunctional photothermal materials that play an important role in solar evaporation and photodegradation. Water purification and desalination methods based on these technologies are both sustainable and scalable due to their ability to absorb sunlight, generate heat, and localize thermal energy near the surface of the water. Their abilities to absorb sunlight, generate heat, and localize thermal energy to the surface of the water make them ideal candidates for sustainable and scalable water purification and desalination methods. Noureen et al. investigated solar-driven steam generation and the decontamination of polluted water using composite hydrogels based on BiVO_4_ and reduced graphene oxide [28]. To our knowledge, there are not many studies that have investigated the photocatalytic activity of modifying BiVO_4_ with MWCNTs [20]. Research is currently limited on MWCNT/BiVO_4_ degradation of antibiotics and their effective role in solar-driven evaporation systems.

**Figure 1:**
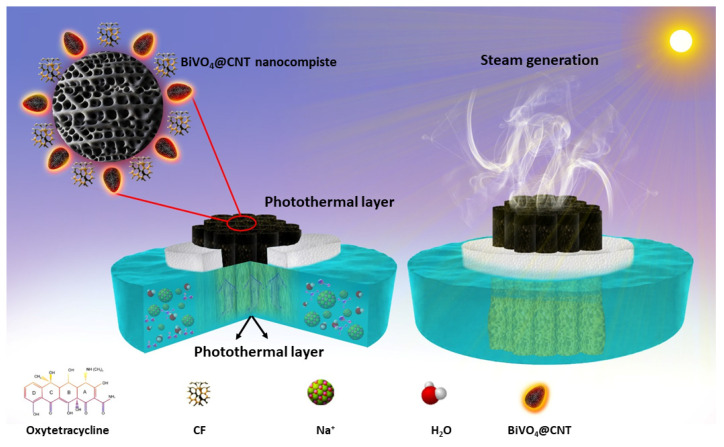
Schematic illustration of the BiVO_4_@CNT-based 3D solar evaporation and photodegradation of OTC.

Herein, we report the preparation of BiVO_4_@CNTs (bismuth vanadate decorated with carbon nanotubes) for highly efficient solar steam generation and photodecomposition of oxytetracycline in source water for freshwater production. First, we synthesized BiVO_4_ nanoparticles (NPs) using the hydrothermal method and then prepared BiVO_4_@CNT nanocomposite via electrostatic reaction mixing. The prepared nanocomposite was coated on assembled cigarette filters to develop an eco-friendly solar evaporation device. The interfacial surface features good solar absorption (95%) and photothermal conversion, excellent hydrophilicity, and optimum thermal management. The findings reveal that the 3D evaporator achieved a good evaporation rate (2.3 kg m^−2^ h^−1^), excellent salt ions rejection capability, and great cycle stability. Additionally, the proposed design reduces environmental stress by exploiting waste objects for useful purposes. More importantly, the BiVO_4_@CNT nanocomposite was tested against the photodegradation of toxic antibiotic oxytetracycline (OTC) in simulated polluted water to assess the photocatalytic potential of the BiVO_4_@CNT nanocomposite. Hence, the present work anticipated multifunctional BiVO_4_@CNT as a potential material for the solar evaporation and photodegradation from wastewater for freshwater production, therefore making a valuable contribution to sustainable development (Figure 1).

## 2. Materials and Methods

### 2.1. Materials

Nitric acid (HNO_3_, GR) was bought from Nanjing Chemical Reagent Co., Ltd. (Nanjing, China) Bismuth nitrate pentahydrate (Bi(NO_3_)_3_·5H_2_O, 99.0%), p-benzoquinone (C_6_H_4_O_2_, 99.5%), sodium orthovanadate (Na_3_VO_4_·12H_2_O, 98.0%), sodium hydroxide (NaOH, 96.0%), dimethyl sulfoxide (C_2_H_6_SO, 99.8%), and carbon nanotubes (CNT) were obtained from the Aladdin Industrial Corporation (Wuhan, China). Ethanol (C_2_H_6_O, AR) was supplied by Sinopharm Chemical Reagent Co., Ltd. (Shanghai, China) All the received chemicals were processed in the experiment without any further purification.

### 2.2. Preparation of BiVO_4_ Nanoparticles

First, 0.1960 g (0.4 mmol) of the solid Bi(NO_3_)_3_·5H_2_O was dissolved into 16 mL glycerol aqueous solution with 25% volume fraction (%) = glycerol × 100%/(glycerol + water) under continuous magnetic stirring for 20 min at room temperature, and the obtained colorless solution was labeled as A. Afterward, the isometric ratio 0.0736 g (0.4 mmol) of Na_3_VO_4_·12H_2_O was dissolved in 16 mL deionized (DI) water while stirring constantly for 20 min, and the obtained transparent solution was labeled as B. Later, solution B was added dropwise into solution A under magnetic agitation for 30 min, and the pH value of the mixture was adjusted between 2 and 6 via HNO_3_ and NaOH solution until the formation of a yellow suspension product, which was labeled as solution C. The obtained yellow suspension was stirred for 20 min and then transferred to the 50 mL PTFE-lined stainless-steel reactor with the addition of an appropriate volume of HNO_3_ solution and NaOH solution (to maintain the pH value) up to an 80% filling degree of the reactor. The reactor was sealed and heated up to 180 °C in a drying oven for 24 h for the sake of reaction completion. The resulting solution was then allowed to cool freely at room temperature and washed over several cycles using DI water and absolute nitrate to remove the undesired impurities. Finally, the washed precursor was placed in a drying oven at 80 °C for 4 h to remove the water. Then, it was ground in the form of fine powder and saved for the further fabrication process.

### 2.3. Synthesis of BiVO_4_@CNT Nanocomposite

The BiVO_4_@CNT nanocomposite was prepared by 2 mmol of Bi(NO_3_)·5H_2_O and dissolved into DI water (50 mL) followed by an addition of MWCNT solution (2 mg, mass fraction 10%, dispersed by 5% PVP 4 mol L^−1^, and HNO_3_ (4 mL) and stirred for half an hour. This solution was labeled as solution X. Afterward, 4 mL of NaOH (2 mol L^−1^) were poured into another beaker and mixed with NH_4_VO_3_ (2 mmol) for half an hour with magnetic stirring until the solution showed a homogeneous texture. The resulting solution was termed solution Y. Then, solutions X and Y were mixed thoroughly, transferred into a Teflon-lined autoclave, and heated for 16 h at 180 °C to complete the reaction. The final product was then centrifuged and washed with ethanol and distilled water several times to remove the undesired species. Finally, the washed product was dried at 70 °C overnight and then calcined in a muffle furnace for 3 h at 400 °C.

### 2.4. Solar Steam Generation Setup

The vapor production experimental process was conducted using a solar simulator (PLS-FX300HU) capable of simulating various sun intensities up to 6 kW m^−2^. A conventional 1.5 GHz amplitude modulation (AM) spectrum was employed in conjunction with an optical filter. The 3D solar evaporator, composed of BiVO_4_/CNT, was placed on the surface of the water (specifically simulated seawater) over a petri dish, ensuring direct contact with the surrounding bulk water. The experimental apparatus was positioned on a sophisticated electronic balance (Mettler Toledo, ME204, Columbus, OH, USA) equipped with a resolution of 0.001 g. This allowed for the measurement of the mass change over time. The setup was subjected to simulated solar radiation, typically at an intensity of 1 kW m^−2^, equivalent to one sun. Following the stability of the entire evaporation system, an assessment was conducted to determine the evaporation rates and maximize the efficiency of solar to vapor conversion under one sun illumination. The study employed an Inductively Coupled Plasma-Optical Emission Spectrometry (ICP-AES) technique, namely the E.P. Optimal 8000 model, to assess and evaluate the levels of salt concentrations in water samples before and after undergoing treatment. All experimental measurements were conducted under ambient environmental conditions, with a temperature of around 29 °C and a humidity level of approximately 43%. Surface temperatures were measured using the Hand-Held Optical Meter Model.

### 2.5. Photocatalytic Experiments

The investigation focused on evaluating the photocatalytic efficiency of the photocatalyst through the examination of the degradation process of oxytetracycline hydrochloride (OTC) antibiotics. This evaluation was conducted under controlled conditions using simulated visible light generated by a Xenon lamp with a power output of 15 W. The experimental procedure involved the illumination of the antibiotic solution in the presence and absence of the BiVO_4_ photocatalyst. The investigation on photodegradation was conducted using an aqueous solution containing an OTC antibiotic at a concentration of 10 ppm and a total volume of 200 cm^3^. A quantity of 50 mg of the BiVO_4_ catalyst was employed. After the process of photo illumination, a sample of 5 cm^3^ was obtained. UV Standard spectrophotometric analysis was used to determine the precise concentration of OTC antibiotics.

## 3. Results

**Figure 2:**
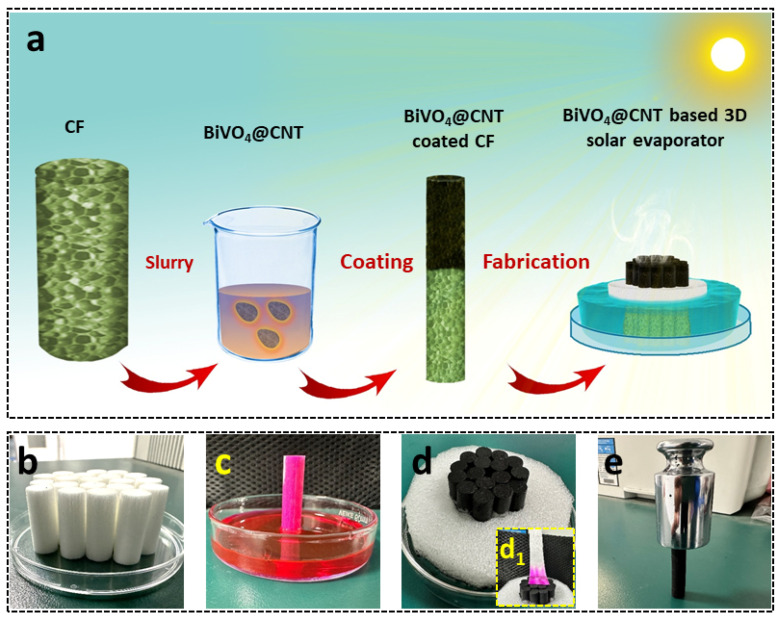
(**a**) Schematic illustration of the facile fabrication of the BiVO_4_@CNT-based 3D solar evaporator; digital images of (**b**) assembled pristine CFs; (**c**) the capillary act of pristine CF; (**d**) the BiVO_4_@CNT-based 3D solar evaporator, (**d_1_**) hydrophilic test; (**e**) the mechanical test of single BiVO_4_@CNT-deposited CF.

The BiVO_4_@CNT solar evaporator was fabricated following a facile fabrication method as schematically illustrated in Figure 2a, which offers promising potential for solar steam generation due to its super hydrophilicity, low thermal conductivity, and good solar absorption. The as-prepared BiVO_4_@CNT composite particles were dissolved into 10% PVA solution and coated on CF by physical coating, which entails the top surface of CF black texture and is capable of absorbing sunlight for photothermal generation. The 3D solar evaporator was constructed using arrays of super-hydrophilic CF arrays inside the PET foam cylindrical substrate to enable floating potential. These cellulosic fibers facilitate the movement of water and efficiently deliver water to the surface via the combined effects of hydrophilicity and capillary force. Figure 2b shows a digital photo of the array of bare cigarette filters assembled in a petri dish. Furthermore, the hydrophilicity of the bare cigarette filter was also investigated by measuring its water absorption potential from the bottom to the top surface as shown in Figure 2c. The investigations confirmed the good hydrophilicity and wettability of cigarette filters as water was quickly wicked up to the top surface of CF when its bottom surface touched the water within 10 s. Hence, a quasi-directional structure composed of cellulose fibers offers good water transportation which is crucial for the efficient replenishment of feedstock for photothermal vaporization. Subsequently, the BiVO_4_@CNT was deposited on the top surface of CF, which imparted its black color, and 16 pieces were assembled into an integrated 3D solar evaporator for solar-driven water evaporation, as shown in Figure 2d, which showed good hydrophilicity for fast evaporation (Figure 2d_1_). Except for hydrophilicity, BiVO_4_@CNT-deposited CF was also checked for mechanical strength by placing 200 N force over a single CF, as shown in Figure 2e. The BiVO_4_@CNT-deposited CF exhibited excellent mechanical robustness under 200 N compression with no surface degradation, revealing its physical durability, endurance, retention, and long-term sustainability for practical implications.

**Figure 3:**
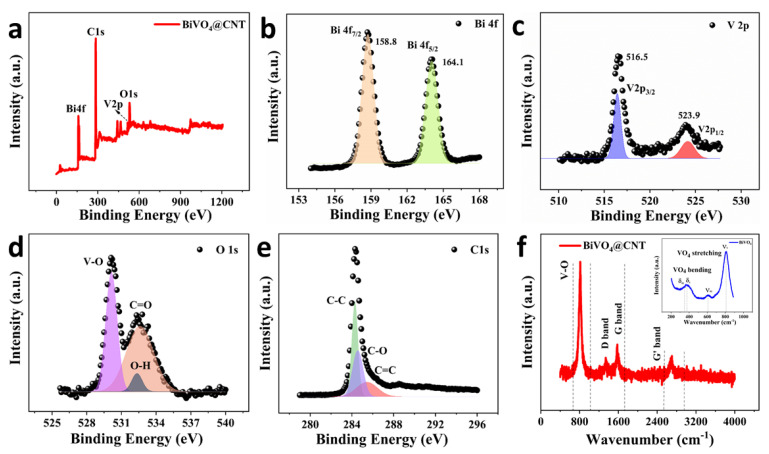
(**a**) XPS spectrum survey of the BiVO_4_@CNT nanocomposite; high-resolution XPS spectrum of (**b**) Bi 4f; (**c**) V 2p; (**d**) O 1s; and (**e**) C1s; (**f**) Raman spectrum of the BiVO_4_@CNT nanocomposite.

Bismuth vanadate BiVO_4_ has shown promising potential as a visible-light photocatalyst for the degradation of hazardous organic effluents from the wastewater discharge of chemical, pharmaceutical, and petrochemical industries. Particularly, BiVO_4_ is reported to have improved photocatalytic activity when it is modified with multi-walled carbon nanotubes (MWCNTs) to develop its heterostructure, owing to the excellent electrical conductivity, large specific surface area, and abundant adsorption sites of MWCNTs [20]. Moreover, CNTs have been investigated extensively as photothermal materials for solar steam generation, owing to their high visible-light absorption efficiency. Hence, CNTs-modified BiVO_4_ offers desirable properties, i.e., high conductivity, good photothermal effect, enhanced photocatalytic performance, significant adsorption potential, and environmental compatibility. The surface elemental composition and chemical bonding of the BiVO_4_@CNT were analyzed using X-ray photoelectron spectroscopy (XPS). Figure 3a shows the whole XPS scan of the BiVO_4_@CNT, revealing the presence of the Bi 4f, C 1s, V 2p, and O 1s elements, respectively.

The high-resolution spectrum of Bi 4f is shown in Figure 3b, which manifests strong symmetrical peaks at 158.8 and 164.1 eV attributed to the Bi 4f_7/2_ and Bi 4f_5/2_ signals account for the 3+ oxidation state of the Bi. Whereas the V 2p spectrum was deconvoluted into two characteristic peaks located at 516.5 and 523.9 eV, which affirms the V 2p_3/2_ and V 2p_1/2_ signals from the 5+ oxidation state of the V (Figure 3c). The lower-binding-energy V 2p_1/2_ component indicates the formation of +4 sates of V, exhibiting that the incorporation of MWCNTs has a shielding effect on bismuth and decreases the electronic density near Bi 4f. The above results are in exact correspondence with the previously reported results of BiVO_4_@CNT heterostructure [20]. The high-resolution spectrum of O1s is shown in Figure 3d, which is deconvoluted of three sub-peaks appearing at 530.14, 532.33, and 532.61 eV attributed to V-O, O-H, and C=O bonds. Figure 3e shows the C1s spectrum, revealing one main peak which was further split into three sub-peaks positioned at 284.28, 284.58, and 285.45 eV binding energies, stipulating the existence of C–C, C–O, and C=O bonds, respectively. BiVO_4_@CNT was also characterized by Raman spectroscopy to measure the vibrational modes of molecules and inner structure as it is an effective tool to characterize carbon-based materials. The Raman spectra of BiVO_4_@CNT are shown in Figure 3f, revealing the characteristic bands of CNTs: a D band (the sp3 defects of carbon atoms) at 1350 cm^−1^, a G band (the sp2-bonded carbon atoms) at 1580 cm^−1^, and a G’ band (D overtone) at 2700 cm^−1^ were observed. The Raman results confirmed that CNT forms a composite with BiVO_4_ [29,30]. Pylarinou et al. [31] also revealed that the most prominent Raman mode at 829 cm^−1^ originates from the symmetric vibration (υ_s_) of V−O stretching, concomitant with the subtle antisymmetric V−O stretching (υ_as_) mode at approximately 710 cm^−1^. Additionally, the symmetric (δ_s_) and antisymmetric (δ_as_) bending modes of the VO_4_ tetrahedra were also identified at 369 and 328 cm^−1^ correspondingly. Meanwhile, the primary external lattice modes manifested at 213 and 129 cm^−1^. Figure 3f (inset) also validates the extended V−O stretching mode.

**Figure 4:**
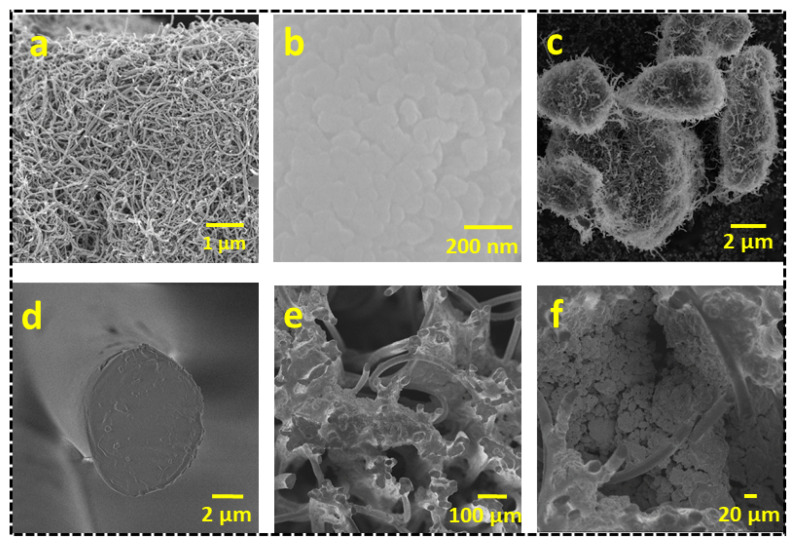
FESEM images of (**a**) Carbon nanotubes (CNT); (**b**) BiVO_4_; (**c**) BiVO_4_@CNT nanocomposite; (**d**) Pristine CF; (**e**,**f**) BiVO_4_@CNT coated CF.

The microstructural and surface morphologies of the CNTs, BiVO_4_, BiVO_4_@CNT, cigarette filter (CF), and BiVO_4_@CNT-deposited CF were analyzed by Field Emission Scanning Electron Microscopy (FESEM). Figure 4a shows the FESEM image of CNTs, revealing the tubular structure of MWCNT at a 1 µm scale with an overall diameter ranging from 50 to 100 nm. Carbon nanotubes (CNTs) possess significant electrical and thermal conductivity properties, with remarkable mechanical strength. The structural stiffness of the carbon bond facilitates the propagation of vibrations throughout the nanotube, hence contributing to its exceptional thermal conductivity. The very high melting point of carbon nanotubes may be attributed to the strong covalent connections formed between each carbon atom, which connects it to three other carbon atoms. Additionally, this phenomenon leads to the presence of an unoccupied electron on each carbon atom, resulting in a sea of delocalized electrons within the tube. Consequently, this arrangement facilitates the conduction of electric current in nanotubes. Figure 4b shows the surface morphology of pure BiVO_4_ nanoparticles, revealing the homogeneous growth. The BiVO_4_ manifested a peanut-shaped microstructure. The formation mechanism of a peanut-shaped structure can be described as the agglomeration of small nanoparticles at the initial stage of hydrothermal treatment under an acidic precursor solution. Afterward, the conglomerated particles developed fully into capsule-like structures through the dissolution and recrystallization processes during heat treatment. Figure 4c represents the morphology of the BiVO_4_@CNT composite, showing the formation of regular-shaped microparticles and rough surface texture to optimize light absorption for an enhanced photothermal and photocatalytic response. The hydrophilic array of cellulose fibers offered enhanced capillary action, resulting in efficient water absorption up to the interfacial surface, hence facilitating vapor formation. Figure 4d shows the FESEM image of the cigarette filter, which manifested the numerous bundles of cellulosic fibers with circular diameters ranging from 20 to 30 µm in a completely random fashion. Water was channeled through the cigarette filter cylinder, which was composed of a fibrous mass that may be disassembled into individual polymer fibers. Water can be supplied rapidly to these hydrophilic threads in the vertical orientation by capillary force and vapor production. Figure 4e shows the morphology of the BiVO_4_@CNT-coated CF, revealing that BiVO_4_@CNT nanocomposites were uniformly distributed over the entire top surface and the texture, giving a dense surface texture. A close-up of a densely coated CF surface with BiVO_4_@CNT strongly embedded in the CF matrix can be seen in the inset of Figure 4f. Because of its intrinsic diffuse reflection feature, the rough morphology of the CF top surface functioned as an efficient photothermal layer, maximizing light capture. Furthermore, good thermal management to accomplish elevated surface temperature confined the converted solar energy at the top contact while limiting its conductivity downward (bulk water).

**Figure 5:**
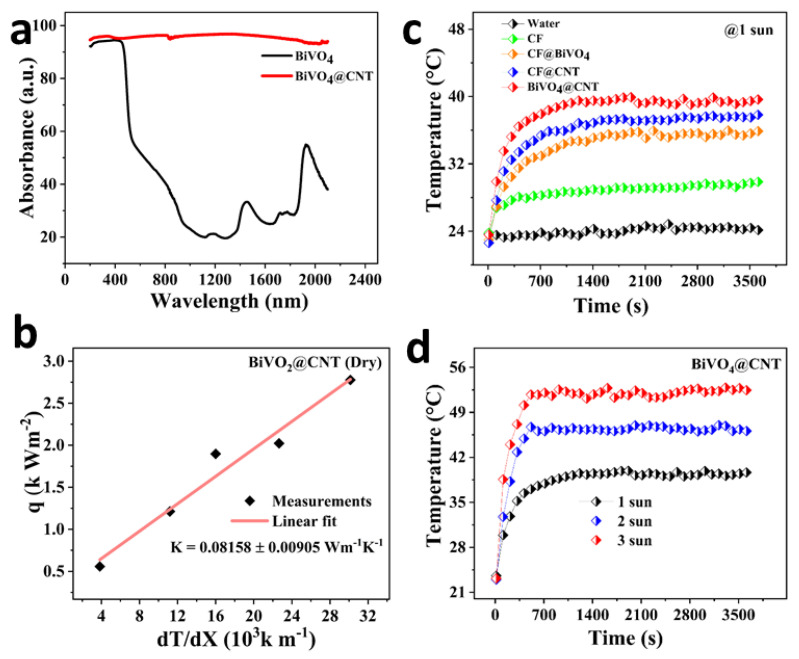
(**a**) UV-vis solar absorption spectrum of the BiVO_4_@CNT nanocomposite powder; (**b**) thermal conductivity of the BiVO_4_@CNT-based 3D solar evaporator; (**c**) surface temperature enhancement of five developed evaporating systems under 1 sun solar irradiance. (**d**) The enhanced surface temperature of the BiVO_4_@CNT-based 3D solar evaporator under different solar irradiations.

The effectiveness of a solar energy-based system is heavily dependent on its ability to efficiently harvest solar light and convert it into photothermal or photochemical energy while minimizing thermal conduction. An efficient 3D solar evaporator was constructed using BiVO_4_@CNT, which demonstrates exceptional solar absorption across the full solar spectral range, together with favorable solar-to-thermal and solar-to-chemical energy conversion efficiencies. The ultraviolet-visible (UV-vis) spectrum ranging from 200 to 2000 nm was obtained for the BiVO_4_@CNT nanocomposite using UV-vis spectroscopy (Figure 5a). The results indicate that the BiVO_4_@CNT nanocomposite demonstrates a high absorption efficiency of 96%. The high absorption capacity seen in this system can be due to the favorable solar absorption and photothermal conversion efficiency of carbon nanotubes (CNTs). These nanomaterials effectively restrict light at the nanoscale, resulting in an extended range of ultrahigh solar absorption that covers the whole solar spectrum.

The 3D solar evaporator possesses a hierarchical porous structure that enables the scattering of incoming light within its dark and porous surface, hence increasing its capacity to capture solar radiation. Effective thermal control has the potential to optimize the efficiency of the entire steam generation system. The thermal conductivity of the BiVO_4_@CNT 3D solar evaporator was determined by experimental measurements utilizing a thermal conductivity meter (Hot Disk, TPS 2500, Sweden hot disk collaboration, Gothenburg, Sweden). Upon activation of the system, a progressive alteration in temperature (*dT/dx*) will manifest in a vertical orientation, resulting in the formation of a temperature gradient. The heat transit rate (*q*) of the BiVO_4_@CNT 3D solar evaporator can be comprehended by employing Fourier equations [32,33].

(1) $$q=-k_{1}\frac{dT}{dx}=-k_{1}\frac{T_{2}-T_{1}}{x_{2}-x_{1}}$$

In the given equation, the symbol *k*_1_ represents the thermal conductivity of the material, which has a value of 1.05 W m^−1^ K^−1^. The symbol *x*_1_ denotes the width of the glass slide, which measures 3 mm. Additionally, *x*_2_ represents the height of the BiVO_4_@CNT evaporator, which is 30 mm. In the context of the thermal conductivity meter, *T*_1_ represents the temperature of the top surface. *T*_2_ and *T*_3_, on the other hand, correspond to the temperatures of the bottom and top surfaces of the glass slides that enclose the BiVO_4_@CNT evaporator, respectively. The thermal conductivity (*k*) of the BiVO_4_@CNT evaporator was determined at the point of temperature equilibrium and sustained rate using the equation provided.

(2) $$k=q\frac{x_{2}}{T_{3}-T_{2}}$$

The BiVO_4_@CNT solar evaporator, as designed, exhibits a notably low thermal conductivity (0.15069 ± 0.00792 Wm^−1^ K^−1^) under dry conditions, as shown in Figure 5b. This can be attributed to the effective scattering of incident light within the interfacial surface, resulting in the transfer of energy as heat to the photothermal surface. Consequently, this leads to a decrease in the thermal conductivity within the overall system’s lower matrix. The rise of interfacial surface temperature, along with little thermal conduction, plays a crucial role in achieving effective thermal management. In this study, we conducted measurements of the surface temperatures of five different developed systems: bulk water, pristine CF, BiVO_4_@CF, CNT@CF, and the BiVO_4_@CNT 3D solar evaporator. The purpose of these measurements was to compare the heat accumulation potential of these systems. To do this, we recorded the surface temperatures under a heat flux of 1 kWm^−2^ for 1 h. Two thermocouples were strategically positioned in the intended regions. The BiVO_4_@CNT 3D solar evaporator demonstrated optimal solar energy capture, efficient distribution of flux throughout the upper matrix, and effective thermal regulation that facilitated water conduction solely toward the top surface while preventing downward heat conduction. The interfacial photothermal surface exhibited a rapid increase in temperature, reaching approximately 40.08 °C. Eventually, it reached an equilibrium temperature for the top surface of the BiVO_4_@CNT solar evaporator, as shown in Figure 5c. This behavior indicates a favorable photothermal conversion rate, as the underlying water surrounds the BiVO_4_@CNT evaporator beneath the interfacial layer. The water was absorbed through a porous assembly and subsequently evaporates through a liquid–gas phase change. The achievement of a high surface temperature facilitated rapid steam generation, resulting in enhanced efficiency. The surface temperature of the BiVO_4_@CNT evaporator was also assessed at varying levels of simulated solar irradiation. It was observed that the highest recorded temperature reached 52.47 °C when subjected to irradiation levels of 3 kWm^−2^ (Figure 5d).

**Figure 6:**
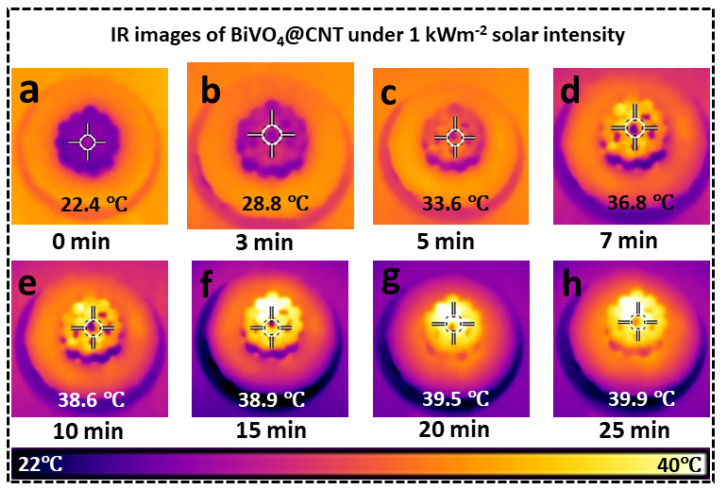
(**a**–**h**) Time-dependent IR images of the BiVO_4_@CNT-based 3D solar evaporator under 1 sun illumination.

In addition, an increase in surface temperature was also observed through the help of an infrared (IR) camera, capturing both top and cross-sectional perspectives, while subjected to a sun intensity of 1 kWm^−2^. The experiment was performed by placing the BiVO_4_@CNT 3D solar evaporator in a petri dish containing water and subjecting it to sun irradiation. The temperature on the upper surface of the evaporator was recorded, as illustrated in Figure 6. Upon activation of the simulated solar intensity, the BiVO_4_@CNT 3D solar evaporator promptly exhibited photothermal conversion on its upper surface. This conversion process involved the absorption and transformation of incident light into thermal energy, increasing the temperature of said surface. According to the data presented in Figure 6a–h, it can be observed that the upper surface of the BiVO_4_@CNT 3D solar evaporator experienced a temperature increase of 38.9 °C within the initial 15 min period, surpassing the surrounding ambient temperature. Within the subsequent 10 min interval, the upper surface was anticipated to attain a state of thermal equilibrium, characterized by a temperature of 39.9 °C. In contrast, the lower matrix of the BiVO_4_@CNT 3D solar evaporator process exhibited a significantly lower temperature compared to the top surface, indicating a lack of heat conduction in the downward direction and effective thermal insulation. This resulted in optimal thermal localization on the top surface. The BiVO_4_@CNT 3D solar evaporator demonstrated an arrangement of anisotropic low thermal conduction and efficient photothermal conversion, resulting in the achievement of an optimal “thermal localization” and, subsequently, a high rate of evaporation. The major aspects considered for the development of a highly efficient steam-generating device are the effective absorption of solar energy, efficient conversion of solar energy into heat, and the hydrophilic properties of the device.

**Figure 7:**
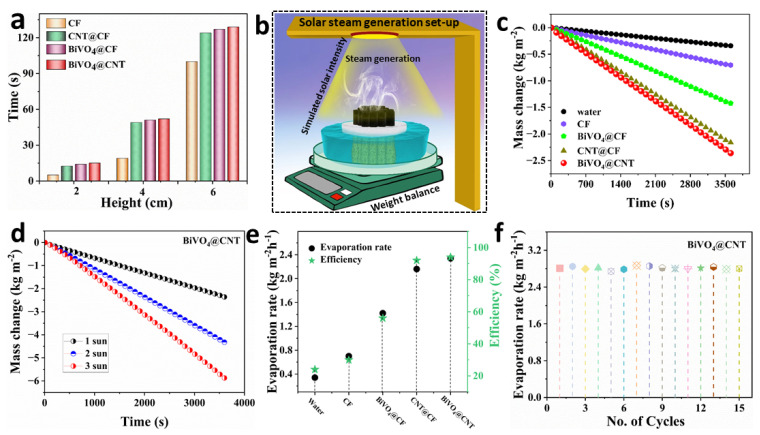
(**a**) The water uptake time for CF, CNT@CF, BiVO_4_@CF, and the BiVO_4_@CF 3D solar evaporator with various heights; (**b**) time-dependent mass variations for the five evaporating systems under one kWm^−2^; (**c**) mass changes of BiVO_4_@CF 3D solar evaporator under multiple solar irradiations; (**d**) schematic illustration of the controlled solar-driven evaporation setup; (**e**) long-term evaporation stability test at 15 cycles; (**f**) evaporation rate and corresponding photothermal conversion efficiency of designed five systems under one sun.

In the context of a solar-driven water evaporation process, the effective and continuous generation of solar steam relies on the essential requirement of rapid water transportation within the evaporator. In Figure 7a, the average water transfer rate is compared among different configurations, namely CF, CNT@CF, BiVO_4_@CF, and BiVO_4_@CNT, at different heights of CF (2, 4, and 6 cm). It is evident that the complete infiltration of a water droplet into the pristine CF occurred within a duration of 4 s. On the other hand, the complete infiltration of a water droplet into BiVO_4_@CNT pristine CF required 15 s. This observation suggests that the hydrophilicity of CF remains intact after the deposition of BiVO4@CNT, facilitating the transfer of water from the bottom to the top surface of the material.

The hydrophilicity of the top surface of the BiVO_4_@CNT solar evaporator, combined with its optimal flux distribution, enables the efficient photothermal conversion of solar light. This is achieved by effectively dispersing the incident solar light at the interface, which serves as the fundamental mechanism for interfacial solar steam generation. In this study, we examined five different evaporation systems: pure water, CF, CNT@CF, BiVO_4_@CF, and BiVO_4_@CNT. The schematic illustration of the experimental set-up of solar steam generation testing is shown in Figure 7b. These systems were analyzed in terms of their continuous vapor generation capabilities under 1 kWm^−2^ irradiation for a duration of 1 h. The objective was to comparatively analyze the evaporation rate and efficiency of each system. It is evident that the high surface temperature, coupled with limited heat conduction, facilitated increased evaporation and a heightened rate of evaporation. The BiVO_4_@CNT solar evaporator demonstrated the highest evaporation rate among the other fabricated systems, namely pure water, CF, BiVO_4_@CF, and CNT@CF. Specifically, the mass change of the BiVO_4_@CNT solar evaporator was recorded at 2.34 kg m^−2^ h^−1^, surpassing the mass change of pure water (0.34 kg m^−2^), CF (0.70 kg m^−2^), BiVO_4_@CF (1.42 kg m^−2^), and CNT@CF (2.16 kg m^−2^), as depicted in Figure 7c. The hydrophilic arrangement of water transport facilitates the rapid and continuous delivery of water to the upper surface, enabling efficient vapor release and effective temperature distribution on said surface. Simultaneously, the internal structure of the hydrogel material facilitates sufficient light penetration to achieve optimal energy absorption and temperature conversion. The BiVO_4_@CNT-based solar evaporator was further tested at different solar intensities to assess its evaporation capabilities under increased solar irradiation, as depicted in Figure 7d. The maximum mass change achieved by BiVO_4_@CNT-based solar evaporator at 3 kWm^−2^ was up to 5.87 kgm^−2^ indicating an enhanced photothermal response when exposed to higher incident light.

The evaporation rate of the BiVO_4_@CNT 3D solar evaporator can be determined by subtracting the evaporation rate under no light conditions from the bulk water. The comparative evaporation rates of these five systems are given in Figure 7e, revealing that the BiVO_4_@CNT solar evaporator achieved the maximum evaporation rate (2.36 kgm^−2^h^−1^), which is higher than the developed four systems and many other reported solar evaporation systems. The photothermal conversion efficiency was calculated using the following equations [34]:

(3) $$\eta_{evap}=\frac{{\dot{m}}_{v}h_{LV}}{q_{solar}}$$

(4) $$h_{LV}=\lambda +C∆T$$

In this equation, *h_LV_* represents the enthalpy change during the liquid-to-vapor phase transition, and λ represents the phase change latent heat during evaporation, specifically referring to the amount of energy required to convert water from a liquid to a vapor phase during photothermal evaporation. The value of λ varies at different temperatures, with a magnitude of 2430 kJ kg^−1^ K^−1^ at 30 °C and 2256 kJ kg^−1^ K^−1^ at 100 °C. The symbol *C* represents the specific heat capacity of water, which is 4.2 kJ kg^−1^ K^−1^. ΔT denotes the temperature difference of water from its initial temperature to the point of vaporization under an irradiance of 1 kWm^−2^, which corresponds to the increase in the surface temperature during photothermal evaporation. Lastly, *q_solar_* represents the simulated solar irradiation, estimated to be up to 1 kWm^−2^ in this context. The studies were conducted under controlled conditions with an ambient temperature of 29 ± 1 °C and a humidity level of 43%. Based on Equations (3) and (4), the calculation of the BiVO_4_@CNT solar evaporator was conducted to determine its evaporation efficiency, which was calculated to be 94%. This calculation excluded any losses due to optical and heat factors. The obtained value of 90% is higher than the evaporation efficiencies of pure water (24%), pure CF (30%), BiVO_4_@CF (56%), and CNT/CF (92%), as depicted in Figure 7e.

Nevertheless, a primary obstacle faced by evaporation structures is the occurrence of structural distortion during prolonged operation, which has a substantial impact on the evaporation rate and efficiency of these devices. In order to investigate the evaporation process without any structural damage, the BiVO_4_@CNT solar evaporator was subjected to multiple operational cycles under one sun, as shown in Figure 7f. The solar evaporator consisting of BiVO_4_@CNT demonstrated consistent evaporation performance, cyclic stability, and durability across 15 evaporation cycles under one sun. The BiVO_4_@CNT solar evaporator had consistent evaporation rates without any notable changes in the evaporation rate during the course of operation, indicating the remarkable stability of the created system. Therefore, we have effectively fabricated a BiVO_4_@CNT solar evaporator that exhibits promising potential for practical implementation without any surface deterioration. This evaporator holds the capability of being deployed on an industrial scale for freshwater generation.

**Figure 8:**
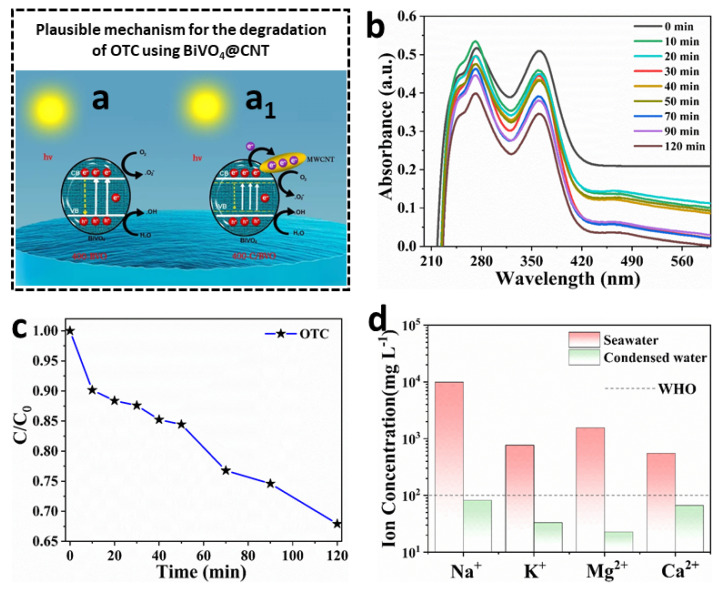
(**a**) Plausible mechanism for the degradation in BiVO_4_ and (**a_1_**) BiVO_4_@CNT; (**b**) the photo-degradation curves of OTC via photocatalysis mechanism; (**c**) normalized concentration of OTC as a function of irradiation time; (**d**) comparative analysis of the concentration of primary metal ions in the simulated seawater and condensed water.

The possible mechanisms of photocatalytic degradation in BiVO_4_@CNT nanocomposite are illustrated in Figure 8. The processes by which BiVO_4_@CNT generated photogenerated electron-hole pairs and the creation of free radicals (•OH and •O^2−)^ were identical. The generation of conduction band electrons (e^−^) and valence band holes (h^+^) occurred upon irradiation of the BiVO_4_@CNT nanocomposite with light energy. Additionally, the production of •OH and •O^2−^ may be characterized by Equations (5) and (6).

(5) $$H_{2}O+h^{+}\to •OH+H^{+}$$

(6) $$O_{2}e^{-}\to •O_{2}^{-}$$

The electrons generated on the BiVO_4_@CNT composite were efficiently transported to the multi-walled carbon nanotubes (MWCNTs) due to the excellent conductivity and electron storage capacity of MWCNTs, as depicted in Figure 8a_1_. The majority of electrons sustained rapid transfer and actively engaged in the reaction between •OH and •O^2−^, resulting in the inhibition of recombination of electron-hole pairs created by light. Nevertheless, in the specific instance of BiVO_4_, the prompt transfer of photogenerated electrons would have been impeded due to a significant proportion of electrons recombining with holes, as studied by Ye et al. [20] (Figure 8a). The introduction of multi-walled carbon nanotubes (MWCNTs) into BiVO_4_ resulted in a decrease in the band gap and hindered the recombination of electron-hole pairs created by light, providing more evidence for the distinct photocatalytic degradation pathways exhibited by BiVO_4_ and BiVO_4_@CNT [20].

In the presence of simulated visible light, tetracycline hydrochloride was utilized as the target material for degradation. Figure 8b illustrates the UV-visible absorption spectra of tetracycline hydrochloride that has undergone degradation using BiVO_4_@CNT. The presented data illustrate a progressive fall in the intensity of the absorption peak at 360 nm over time. Similarly, the peak at 270 nm also exhibited a gradual decrease, accompanied by a noticeable blue shift. In addition, it can be observed from Figure 8c that the degradation rate of OTC by BiVO_4_@CNT reached 74.3% after 120 min of photocatalysis. The findings of the study suggest that BiVO_4_@CNT exhibits the highest level of photocatalytic activity. In the current study, the BiVO_4_@CNT composite exhibited a heightened photoactivity of 83% regarding the breakdown of the OTC antibiotic. This finding suggests that the photocatalyst exhibits good efficacy in the removal of antibiotics from wastewater exploiting the cost-effective solar energy.

Multiple investigations were conducted in order to enhance the multimodal rejection capabilities of the BiVO_4_@CNT solar evaporator and maintain its quality of producing clean water. The interconnected polymeric network plays a crucial role in facilitating the evaporation rate through molecular mechanisms. Additionally, it significantly improves the purification efficiency by effectively removing main salt and heavy metal ions. Figure 8d shows the results of inductively coupled plasma atomic emission spectroscopy (ICP-OES) measurements of the concentration of four important metal ions in stimulated seawater and condensed water. These ions were sodium (Na^+^), potassium (K^+^), magnesium (Mg^2+^), and calcium (Ca^2+^). With the condensation process, the metal ion level of the water dropped dramatically, far below the standards for safe drinking water set by the WHO and the EPA in the United States. The capacity to generate drinkable water from seawater is key to the viability of the interfacial BiVO_4_@CNT solar evaporator for desalination. Therefore, we have successfully fabricated a highly efficient solar evaporator consisting of 3D BiVO_4_@CNT, which holds promise for practical implementation. This device exhibits excellent metal ion rejection capabilities without any surface deterioration, making it suitable for large-scale industrial deployment in freshwater production. CNTs should be loaded in the composite in the most optimal manner possible. It is crucial to determine the right balance between the amount of CNTs and BiVO_4_ to maximize photothermal performance and achieve synergistic effects [35,36]. Insufficient interaction between CNTs can result in insufficient charge transfer, whereas excessive CNT concentration could result in aggregation and decreased light absorption. CNT loading and analysis of the composite’s structural and functional properties should be the focus of optimization studies aimed at producing the highest photothermal ability. Table 1 demonstrates the optimization of the CNT loading and its significant impact on evaporation performance. It is noteworthy that BiVO_4_ is particularly compelling for use as a photocatalyst since it is capable of harnessing visible light, which accounts for a substantial amount of solar energy [20]. Following the global endeavor to develop environmentally friendly and energy-efficient technologies, BiVO_4_ offers a distinct advantage for sustainable and energy-efficient wastewater treatment operations [20,21,22]. One specific avenue of investigation emerging from the findings is the enhancement of BiVO_4_’s photocatalytic activity by modification with multi-walled carbon nanotubes (MWCNTs) [20]. This modification results in a heterostructure that takes advantage of the extremely high electrical conductivity, large specific surface area, and numerous adsorption sites of MWCNTs. By combining the photocatalytic abilities of MWCNTs with the properties of BiVO_4_, the composite material is capable of degrading organic pollutants under visible illumination.

## 4. Conclusions

In summary, an efficient 3D solar steam generation was reported using a BiVO_4_@CNT nanocomposite which was coated over CF for the development of a sustainable and environmentally friendly steam generating system. The CF showed good hydrophilicity and superior solar absorption (96%) due to BiVO_4_@CNT coating deposition on the top surface, which promoted heat confinement on the upper matrix with minimum thermal conduction to underlying water. The BiVO_4_@CNT-based 3D solar evaporator achieved enhanced evaporation rates (2.36 kg m^−2^ h^−1^) under 1 kW m^−2^ solar irradiation with a corresponding photothermal conversion efficiency of up to 94%, while successfully rejecting the metal ions from seawater. Additionally, the BiVO_4_@CNT nanocomposite also showed increased photoactivity up to 83% of the photodegradation of oxytetracycline (OTC) antibiotics from industrial and pharmaceutical wastewater, which expands its application for freshwater production from chemical and industrial water. From the performance of the developed system, we believe that the BiVO_4_@CNT 3D solar evaporator can offer a simple and long-lasting method for producing potable water from the sea and industrial wastewater to address the ever-increasing water scarcity challenges.

## Figures and Tables

**Table 1:** The optimization of CNT loading concentration and its impact on photothermal conversion efficiency.

Sr No.	CNTs Concentrations	Evaporation Rate (kg m^−2^h^−1^)	Photothermal Conversion Efficiency (%)
1.	1 mg	2.31	89%
2.	2 mg	2.36	92%
3.	3 mg	2.33	90.2%

## Data Availability

The data will be furnished upon reasonable request.

## References

[1] Irshad M.S., Arshad N., Asghar M.S., Hao Y., Alomar M., Zhang S., Zhang J., Guo J., Ahmed I., Mushtaq N. (2023). Advances of 2D-Enabled Photothermal Materials in Hybrid Solar-Driven Interfacial Evaporation Systems toward Water-Fuel-Energy Crisis. Adv. Funct. Mater..

[2] Seckler D., Barker R., Amarasinghe U. (1999). Water Scarcity in the Twenty-First Century. Int. J. Water Resour. Dev..

[3] Liu J., Yang H., Gosling S.N., Kummu M., Flörke M., Pfister S., Hanasaki N., Wada Y., Zhang X., Zheng C. (2017). Water Scarcity Assessments in the Past, Present, and Future. Earth’s Futur..

[4] LI Z., QI W., Yao F., LIU Y., Ebrahim S., Jian L. (2019). Degradation Mechanisms of Oxytetracycline in the Environment. J. Integr. Agric..

[5] Yang Y., Zeng G., Huang D., Zhang C., He D., Zhou C., Wang W., Xiong W., Li X., Li B. (2020). Molecular Engineering of Polymeric Carbon Nitride for Highly Efficient Photocatalytic Oxytetracycline Degradation and H_2_O_2_ Production. Appl. Catal. B Environ..

[6] Wei Z., Arshad N., Hui C., Irshad M.S., Mushtaq N., Hussain S., Shah M., Naqvi S.Z.H., Rizwan M., Shahzad N. (2022). Interfacial Photothermal Heat Accumulation for Simultaneous Salt Rejection and Freshwater Generation; an Efficient Solar Energy Harvester. Nanomaterials.

[7] Boretti A., Rosa L. (2019). Reassessing the Projections of the World Water Development Report. npj Clean Water.

[8] Chen R., Zhu K., Gan Q., Yu Y., Zhang T., Liu X., Ye M., Yin Y. (2017). Interfacial Solar Heating by Self-Assembled Fe_3_O_4_@ C Film for Steam Generation. Mater. Chem. Front..

[9] Huang W., Hu G., Tian C., Wang X., Tu J., Cao Y., Zhang K. (2019). Nature-Inspired Salt Resistant Polypyrrole–Wood for Highly Efficient Solar Steam Generation. Sustain. Energy Fuels.

[10] Ghafurian M.M., Niazmand H., Akbari Z., Bakhsh Zahmatkesh B. (2019). Performance Evaluation of Ferric Oxide (Fe_3_O_4_) and Graphene Nanoplatelet (GNP) Nanoparticles in Solar Steam Generation. J. Solid Fluid Mech..

[11] Irshad M.S., Arshad N., Liu G., Mushtaq N., Lashari A.A., Qin W., Asghar M.S., Li H., Wang X. (2023). Biomass-Printed Hybrid Solar Evaporator Derived from Bio-Polluted Invasive Species, a Potential Step toward Carbon Neutrality. ACS Appl. Mater. Interfaces.

[12] Liu G., Yu F., Irshad M.S., Xiong X., Guo Z., Wang J., Xiao B., Lin L., Wang X. (2022). Biomass-Inspired Solar Evaporator for Simultaneous Steam and Power Generation Enhanced by Thermal-Electric Effect. Energy Technol..

[13] Tao P., Ni G., Song C., Shang W., Wu J., Zhu J., Chen G., Deng T. (2018). Solar-Driven Interfacial Evaporation. Nat. Energy.

[14] Zhu M., Li Y., Chen F., Zhu X., Dai J., Li Y., Yang Z., Yan X., Song J., Wang Y. (2018). Plasmonic Wood for High-Efficiency Solar Steam Generation. Adv. Energy Mater..

[15] Ahmad Wani T., Garg P., Bera S., Bhattacharya S., Dutta S., Kumar H., Bera A. (2022). Narrow-Bandgap LaMO_3_ (M = Ni, Co) Nanomaterials for Efficient Interfacial Solar Steam Generation. J. Colloid Interface Sci..

[16] Jiang H., Ai L., Chen M., Jiang J. (2020). Broadband Nickel Sulfide/Nickel Foam-Based Solar Evaporator for Highly Efficient Water Purification and Electricity Generation. ACS Sustain. Chem. Eng..

[17] Yang M.-Q., Tan C.F., Lu W., Zeng K., Ho G.W. (2020). Spectrum Tailored Defective 2D Semiconductor Nanosheets Aerogel for Full-Spectrum-Driven Photothermal Water Evaporation and Photochemical Degradation. Adv. Funct. Mater..

[18] Shi L., Wang X., Hu Y., He Y., Yan Y. (2020). Solar-Thermal Conversion and Steam Generation: A Review. Appl. Therm. Eng..

[19] Bai W., Zhou Y., Peng G., Wang J., Li A., Corvini P.F.-X. (2022). Engineering Efficient Hole Transport Layer Ferrihydrite-MXene on BiVO4 Photoanodes for Photoelectrochemical Water Splitting: Work Function and Conductivity Regulated. Appl. Catal. B Environ..

[20] Ye S., Zhou X., Xu Y., Lai W., Yan K., Huang L., Ling J., Zheng L. (2019). Photocatalytic Performance of Multi-Walled Carbon Nanotube/BiVO4 Synthesized by Electro-Spinning Process and Its Degradation Mechanisms on Oxytetracycline. Chem. Eng. J..

[21] Li Y., Liu Y., Xing D., Wang J., Zheng L., Wang Z., Wang P., Zheng Z., Cheng H., Dai Y. (2021). 2D/2D Heterostructure of Ultrathin BiVO_4_/Ti_3_C_2_ Nanosheets for Photocatalytic Overall Water Splitting. Appl. Catal. B Environ..

[22] Madhusudan P., Ran J., Zhang J., Yu J., Liu G. (2011). Novel Urea Assisted Hydrothermal Synthesis of Hierarchical BiVO_4_/Bi_2_O_2_CO_3_ Nanocomposites with Enhanced Visible-Light Photocatalytic Activity. Appl. Catal. B Environ..

[23] Yu J., Kudo A. (2006). Effects of Structural Variation on the Photocatalytic Performance of Hydrothermally Synthesized BiVO4. Adv. Funct. Mater..

[24] Das S., Ahn Y.-H. (2022). Preparation of P-Doped CdS Nanorods as an Efficient Photocatalyst for the Degradation of the Emerging Pollutant Tetracycline Antibiotic under Blue LED Light Irradiation. Dalt. Trans..

[25] Boxall A.B.A. (2004). The Environmental Side Effects of Medication: How Are Human and Veterinary Medicines in Soils and Water Bodies Affecting Human and Environmental Health?. EMBO Rep..

[26] Karim A.V., Krishnan S., Pisharody L., Malhotra M., Bustillo-Lecompte C. (2020). Application of Ferrate for Advanced Water and Wastewater Treatment. Adv. Oxid. Process..

[27] Irshad M.S., Arshad N., Wang X. (2021). Nanoenabled Photothermal Materials for Clean Water Production. Glob. Chall..

[28] Noureen L., Xie Z., Hussain M., Li M., Lyu Q., Wang K., Zhang L., Zhu J. (2021). BiVO4 and Reduced Graphene Oxide Composite Hydrogels for Solar-Driven Steam Generation and Decontamination of Polluted Water. Sol. Energy Mater. Sol. Cells.

[29] Melvin G.J.H., Ni Q.-Q., Suzuki Y., Natsuki T. (2014). Microwave-Absorbing Properties of Silver Nanoparticle/Carbon Nanotube Hybrid Nanocomposites. J. Mater. Sci..

[30] Prasad U., Prakash J., Azeredo B., Kannan A. (2019). Electrochemical Investigation of the Effect of Graphitic Carbon Nitride Addition in BiVO_4_ to Improve Photoelectrochemical Water Oxidation Performance. Abstracts of Papers of the American Chemical Society.

[31] Pylarinou M., Sakellis E., Tsipas P., Romanos G.E., Gardelis S., Dimoulas A., Likodimos V. (2023). Mo-BiVO_4_/Ca-BiVO_4_ Homojunction Nanostructure-Based Inverse Opals for Photoelectrocatalytic Pharmaceutical Degradation under Visible Light. ACS Appl. Nano Mater..

[32] Irshad M.S., Wang X., Abbasi M., Arshad N., Chen Z., Guo Z., Yu L., Qian J., You J., Mei T. (2021). Semiconductive, Flexible MnO_2_ NWs/Chitosan Hydrogels for Efficient Solar Steam Generation. ACS Sustain. Chem. Eng..

[33] Arshad N., Irshad M.S., Asghar M.S., Alomar M., Tao J., Shah M.A.K.Y., Wang X., Guo J., Wageh S., Al-Hartomy O.A. (2023). 2D MXenes Embedded Perovskite Hydrogels for Efficient and Stable Solar Evaporation. Glob. Chall..

[34] Xiao Q., Zhu Y., Xi Y., Kong X., Ye X., Zhang Z., Qiu C., Xu W., Cheng S., Zhang J. (2022). Highly Charged Hydrogel with Enhanced Donnan Exclusion toward Ammonium for Efficient Solar-Driven Water Remediation. Chem. Eng. J..

[35] Guo Y., Han W., Zhao K., Hao S., Shi S., Ding Y. (2022). Promoting Effects of Y Doping and FeOOH Loading for Efficient Photoelectrochemical Activity on BiVO_4_ Electrodes. New J. Chem..

[36] Orimolade B.O., Arotiba O.A. (2020). Bismuth Vanadate in Photoelectrocatalytic Water Treatment Systems for the Degradation of Organics: A Review on Recent Trends. J. Electroanal. Chem..

